# Predicting Factors for Rapid Progressive Chronic Kidney Disease in Primary Glomerular Disease Patients with Moderate-to-Severe Stage

**DOI:** 10.1155/2020/3796792

**Published:** 2020-11-28

**Authors:** Jun Tan, Hao Zhang, Wei Zheng, Shikun Yang, Minghui Yang, Yan Liu

**Affiliations:** Department of Nephrology, The Third Xiangya Hospital, Central South University, Changsha, Hunan, China

## Abstract

**Objective:**

To investigate the predictive factors associated with rapid progressive chronic kidney disease (CKD) in patients with primary glomerular disease (PGD).

**Methods:**

Baseline data, clinical biochemistry, laboratory data, and imaging data were collected from 112 PGD patients in CKD stages 3 and 4 who were hospitalized at the Third Xiangya Hospital. Patients were divided into rapid progression group (Group R) and no rapid progression group (NR) according to the definition of rapid progression of CKD.

**Results:**

The age, systolic blood pressure (SBP), serum *β*2-microglobulin (s*β*2-MG), urinary *α*1-microglobulin (u*α*1-MG), and cardiothoracic ratio (CTR) of the R group were significantly higher than the NR group. However, the size of the kidney, high-dense lipoprotein (HDL), hemoglobin (Hb), and hematocrit of the R group were significantly lower than the NR group (*P* < 0.05). Binary logistic regression analysis showed that baseline CTR, SBP, size of the kidney, and HDL were independent risk factors for rapid progression of PGD. At the end of follow-up, CTR and SBP of group R were higher than the NR group, and the size of the kidney and HDL of group R were lower than the NR group.

**Conclusion:**

Increased baseline CTR and SBP and decreased baseline HDL and renal volume could be the predictors of rapid progression in patients of PGD at the CKD stages 3 and 4.

## 1. Introduction

A meta-analysis of 100 articles indicated that the average global prevalence rate of chronic kidney disease (CKD) is about 13.4%, among which the prevalence rate of CKD stages 1-5 is 3.5%, 3.9%,7.6%, 3.5%, and 0.1%, respectively [1]. The majority of CKD patients are in stages 3 and 4. Primary glomerular disease (PGD) is a kind of primary glomerular disease characterized by hematuria, proteinuria, edema, and hypertension. PGD is one of the common causes of end-stage renal disease (ESRD). ESRD patients would need renal replacement treatment, which brings patients and society economical and psychological burden. Previous studies showed that severe infection, acute heart failure, urinary tract obstruction, and hypovolemic shock may lead to acute-on-chronic renal injury and hyperuricaemia [2, 3]. High protein diet, genetic, and epigenetic variants are risk factors of rapid progression of CKD [4, 5]. Coresh et al. found that a 30% decline of estimated glomerular filtration rate (eGFR) within 2 years was associated with about 5-fold increased risk of ESRD after adjustment for covariates including baseline eGFR [3]. However, up to now, no biomarkers are available as predictors of rapid progression of CKD caused by PGD, which can help physicians perform more strict education and rigorous treatment in patients at the initial stage to avoid the unexpected consequence.

Therefore, this study is aimed at identifying risk factors and predictors of rapid progression of CKD caused by PGD. Our results help establish a diagnostic standard of rapid progression of PGD with high sensitivity and specificity.

## 2. Materials and Methods

### 2.1. Patients

This study was approved by the Ethics Committee of Third Xiangya Hospital, and no informed consent was required because this is a retrospective study. A total of 306 PGD patients were screened who were hospitalized at the nephrology department in the Third Xiangya Hospital from January 2014 to September 2016 and initially diagnosed as chronic CKD stages 3-4 caused by PGD. Patients were excluded if they met the exclusion criteria: (1) patients who have been treated with renal replacement therapy (*n* = 11); (2) patients suffering severe infection, cardiovascular, cerebrovascular disease, liver failure, and respiratory failure (*n* = 63); (3) patients using hormones or immunosuppressants (*n* = 15); (4) patients who did not participate regularly follow-up examination for 2 years (*n* = 61); (5) Patients with missing data > 20% (*n* = 44). Finally, 112 eligible patients were included in this study.

### 2.2. Diagnosis

The standard diagnosis of PGD conformed to the definition of 2012 KDIGO guidelines for glomerulonephritis. The standard diagnosis of hypertension: SBP ≥ 130 mmHg and (or) diastolic blood pressure (DBP) ≥ 80 mmHg according to America College of Cardiology/American Heart Association in 2017. The standard diagnosis of diabetes: the history of diabetes, fast plasma glucose (FPG) ≥ 7.0 mmol/L, or 2 hours postprandial blood glucose (PBG) ≥ 11.1 mmol/L, or random blood glucose (RBG) ≥ 11.1 mmol/L. The standard diagnosis of rapid progressive primary glomerular disease (PGD): reduction in eGFR of at least 30% from baseline within 2 years and/or commencement of dialysis.

### 2.3. Data Collection

The baseline data were collected from the patients, including age, sex, body mass index (BMI), basal metabolic rate (BMR), mean arterial pressure (MAP), size and lesion of parenchyma of kidney testing by ultrasound, cardiothoracic ratio (CTR) obtained by chest X-ray and serum indicators such as hemoglobin (Hb), creatinine (Cr), blood urea nitrogen (BUN), potassium (K), chlorine (Cl), albumin (Alb), total cholesterol (TC), cystatin C (CysC), factor B, homocysteine (Hcy), ceruloplasmin (CP), and urinary indicators such as uACR, u*β*2-MG, and uRBP. The eGFR was calculated with the formula modified for Chinese: GFR = 169 × (Cr/88.4)^−0.608^ × CysC^−0.63^ × Age^−0.157^(×0.83 if female) [6]. The parenchyma, the length, and diameter of the kidney were evaluated by B-ultrasound. We categorized renal parenchyma into I to IV: the renal parenchyma density less than liver (I), the renal parenchyma density is equal to liver (II), the renal parenchyma density slightly higher than liver (III), and the renal parenchyma density higher than liver (IV). CTR was classified into three levels: 0 (CTR < 0.52), 1 (0.52 ≤ CTR < 0.55), 2 (0.55 ≤ CTR < 0.6), and 3 (CTR ≥ 0.6). Over 30% reduction of eGFR in 2 years was chosen as an endpoint for the rapid progression of CKD caused by PGD, and the patients were divided into rapid progression group (R) and no rapid progression group (NR). The standards of the acceptable blood pressure (BP) and hemoglobin in PGD patients were as follows: BP ≤ 130/80 mmHg, and Hb ≥ 130 g/L in male (or 120 g/L in female) according to KDIGO guidance on anemia.

### 2.4. Statistical Analysis

Data in normal distribution were expressed as mean ± SD and compared by ANOVA and *t*-test. Data not in normal distribution were expressed as median (25th to 75th percentiles) and compared by nonparametric test (Wilcoxon rank-sum test). Qualitative variables were expressed as percentages and compared by the Chi-square test. Logistic regression analysis was used to identify risk factors and predictors. *P* < 0.05 indicated statistical significance.

## 3. Results

A total of 112 patients (average age of group NR and group R were 51.62 ± 16.68 and 58.27 ± 12.92 years, respectively) with CKD caused by PGD participated in the study, including 60 males and 52 females. According to the stage of CKD, there were 52 patients in stage 3 and 60 patients in stage 4. After the 2-year follow-up, there were 18 patients in stages 1-2, 21 patients in stage 3, 35 patients in stage 4, and 38 patients in stage 5, which indicated that most patients have entered into end-stage within 2 years.

Univariate analysis showed that age, SBP, s*β*2-MG, u*α*2-MG, and CTR were significantly higher in the R group than in the NR group, while the kidney size, HDL, Hb, and Hct were significantly lower in the R group than in the NR group (*P* < 0.05). Other factors showed no significant differences between the two groups such as diabetes history, CRP, LDL-C, TC, TG, ESR, CP, prealbumin (PA), Alb, ferritin, transferrin, *α*1-MG, RBP, serum *β*-d-glucosaminidase (sNAG), and urinary *β*-d-glucosaminidase (uNAG) (Tables 1 and 2).

**Figure 1:**
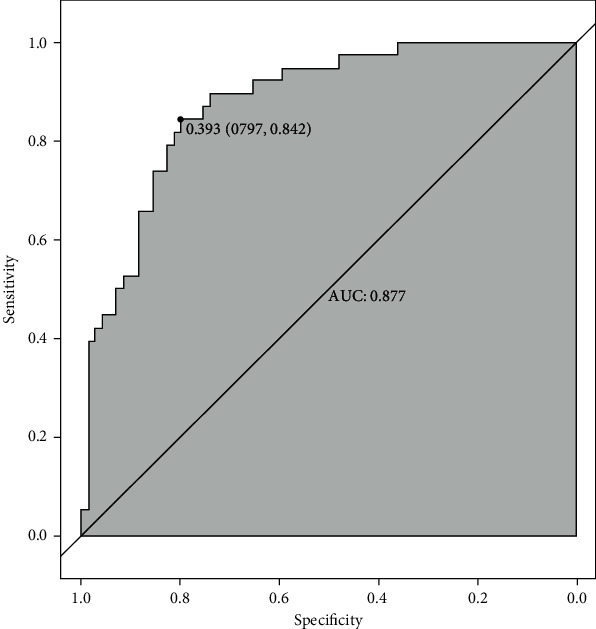
ROC curve of independent risk factors.

Binomial logistic regression analysis revealed that CTR, SBP, kidney size, and HDL were independent risk factors for the rapid progression of PGD (Table 3). The standard regression formula was logit(P) = 1.559 × CTR + 0.042 × SBP − 1.620 × HDL − 0.007 × (kidney size) − 10.296 (*P* < 0.001, *R*^2^ = 0.401). To test the predictive performance of the regression formula, we constructed a receiver operating characteristic (ROC) curve (Figure 1). The area under the ROC curve (AUC) was 0.877, and the specificity and sensitivity were 0.797 and 0.842, respectively.

After the 2-year follow-up, we found that SBP and CTR of the R group were significantly higher than baseline, while renal volume and HDL were lower than baseline (*P* < 0.05). However, such changes were not observed in the NR group. At the end of the follow-up, CTR and SBP were significantly higher, and HDL was significantly lower in the R group than in the NR group (Table 4).

In different CKD stages, the change of corresponding indicators was observed (Table 5). In the patients with CKD stage 3, CTR, and SBP at the endpoint were significantly higher than the baseline (*P* < 0.05), HDL significantly decreased compared with the baseline, and the baseline and renal volume of follow-up cases did not show significant differences. In patients with CKD stage 4, the follow-up endpoints CTR and SBP increased significantly, and the renal volume and HDL decreased significantly compared to the baseline value (*P* < 0.05).

At the baseline, BP reaching threshold (BP < 130/80 mmHg) was about 34%. At the endpoint, BP reaching threshold was 25.3%. The qualified rate of BP control in the R group dropped from 20% to 13%.

## 4. Discussion

It is generally acknowledged that CTR directly reflects cardiac size, relating to cardiac failure [7, 8]. This retrospective cohort study is aimed at exploring biomarkers as the predictors of rapid progression of CKD caused by PGD. For the first time, we found that CTR > 0.5 was the risk factor and predictor of the rapid process of CKD caused by PGD in the patients with CKD stages 3-4. The increase of CTR correlates with age, BMI, coronary artery stenosis, and impairment of left ventricular function [7]. CTR was significantly related to target organ injury in patients with hypertension [8]. A large sample study on the CKD population showed that all-cause mortality occurred in 28.5% of patients with normal CTR (≤0.50) and 34.3% of patients with high CTR (>0.50) [9]. Compared with baseline values, we found that more patients with PGD had increased CTR at the endpoint. It may correlate with the accumulation of toxins, anemia, and unmanageable blood pressure [10]. The mechanism needs further investigation. Therefore, CTR is critical for patients with CKD caused by PGD, and it is necessary to constantly monitor CTR to facilitate timely intervention.

Some studies suggested that hypertension with renal failure is more difficult to control, and the risk of cardiovascular disease significantly increases [11, 12]. Indeed, SBP is an independent risk factor of the rapid progress of CKD. Compared with DBP, SBP shows a stronger ability in predicting the occurrence of ESRD events [13, 14]. With the progress of CKD, increased salt and water retention, excessive activation of the renin-angiotensin-aldosterone system, and higher level of sympathetic activation with decreasing eGFR would contribute to uncontrolled SBP [15, 16]. Our study revealed that SBP is the risk factor and predictor of the rapid progress of CKD caused by PGD. At the endpoint, the SBP of patients in the R group was higher than the baseline, and the qualified rate of BP control in the R group dropped from 20% to 13%. It was reported that CKD would rapidly progress when SBP < 110 mmHg, especially in the later period of CKD without albuminuria [17]. Therefore, it is necessary to require patients to monitor their blood pressure regularly, control the lifestyle, and take an individualized antihypertensive approach.

Current evidence indicates that dyslipidemia is closely related to the occurrence and progress of CKD. The mechanism of abnormal blood lipids affecting kidney diseases is manifold and is related to oxidative stress, proteinuria, and lipoprotein transport disorders [18]. In kidney diseases, dyslipidemia is usually manifested in elevated TG, diminished HDL, and elevated LDL. HDL-C has antioxidant and anti-inflammatory effects [19]. In CKD patients, HDL reduced cholesterol efflux capacity in macrophages, the ability of antioxidant, and anti-inflammatory actions [20]. Moreover, lipolysis efficiency decreased with a reduction in eGFR [21]. Kawachi et al. found that a low serum HDL-C level could be a significant predictor of CKD progression, especially in female patients with CKD under 70 years of age [22]. Bowe et al. reported that compared to those with HDL-C of 40 mg/dl or more, low HDL-C (under 30 mg/dl) was associated with the increased risk of incident eGFR under 60 ml/min/1.73 m [23]. Our study showed that decreased baseline HDL was the predictor of rapid progression of CKD3-4 caused by PGD.

Renal function may decline rapidly with parenchymal lesions and nephrons loss. In addition, the kidney size is proportional to the number of nephron and is significantly associated with renal function [24]. Therefore, the decline in renal function can be judged to some extent by the change in kidney size [25]. In our study, we observed that baseline renal volume was the independent predictor of rapid progression of PGD during two years by using body surface area to correct the size of the kidney.

In addition, there were 18 patients in stages 1 and 2 after the 2-year follow-up. This is a remarkable finding. There is a possibility of renal function recovery in the early stages of disease, or there are some factors that affect the SCr and eGFR when they are tested, such as diet, exercise, and medicine. Our study has some limitations. While we showed that 4 parameters (CTR, SBP, HDL, and renal volume) could be the predictors of rapid progression of PGD, it remains to decide whether the presence of a single parameter is important or complete the set of 4 parameters is indispensable for the prediction.

In conclusion, increased baseline CTR and SBP and decreased baseline HDL and renal volume could be the predictors of rapid progression of PGD in patients of PGD at the CKD stages 3 and 4.

## Figures and Tables

**Table 1:** Clinical characteristics of the study population at baseline.

Indicators		Group NR (*n* = 70)	Group R (*n* = 42)	*P*
Age (years)		51.62 ± 16.68	58.27 ± 12.92	0.031
Gender	Female	32 (45.7%)	20 (47.6%)	0.95
	Male	38 (54.3%)	22 (52.4%)	
BMI (kg/m^2^)		23.01 ± 3.48	23.68 ± 3.95	0.36
Blood pressure	SBP (mmHg)	129.96 ± 17.42	140.15 ± 17.01	0.002
	DBP (mmHg)	78.69 ± 11.0	79.12 ± 11.07	0.85
	MAP (mmHg)	95.87 ± 12.22	99.51 ± 11.61	0.13
Antihypertensive drugs	No-antihypertensive treatment	45 (64.3%)	26 (61.9%)	0.94
	Antihypertensive treatment	25 (35.7%)	16 (38.1%)	
Diabetes history	No	64 (91.4%)	33 (78.6%)	0.09
	Yes	6 (8.6%)	9 (21.4%)	

**Table 2:** Laboratory and imaging characteristics of the study population at baseline.

Indicators		Group NR (*n* = 70)	Group R (*n* = 42)	*P*
Renal function	Baseline eGFR (mL/min·1.73 m^2^)	31.05 (16.01, 41.18)	23.35 (17.40, 41.64)	0.80
	*Δ*eGFR (mL/min·1.73 m2)	1.466 (-4.738, 4.646)	15.302 (8.824, 22.694)	0.000
	CysC (mg/L)	1.99 ± 0.75	2.24 ± 1.22	0.17
	UA (mmol/L)	455.08 ± 127.31	442.57 ± 117.2	0.60
Blood routine, urine routine	WBC (∗10^12^/L)	7.7 ± 2.84	7.81 ± 3.34	0.86
	Hb (g/L)	114.53 ± 24.47	105.72 ± 27.7	0.014
	Hct (%)	34.75 ± 7.03	31.45 ± 6.97	0.020
	uSG	1.02 (1.015, 1.02)	1.02 (1.015, 1.02)	0.80
	uPH	5.5 (5.0, 6.0)	6 (5.38, 6.50)	0.62
Lipid	TC (mmol/L)	5.21 (4.06, 6.41)	4.40 (3.83, 5.56)	0.19
	TG (mmol/L)	1.71 (1.0, 2.66)	1.63 (1.28, 2.57)	0.74
	LDL-C (mmol/L)	2.64 (2.06, 3.37)	2.28 (1.97, 2.82)	0.37
	HDL-C (mmol/L)	1.28 (0.97, 1.62)	1.2 (0.90, 1.37)	0.024
Electrolyte	K (mmol/L)	4.04 ± 0.57	4.14 ± 0.7	0.43
	Na (mmol/L)	140 ± 3.26	140.01 ± 4.09	0.98
	Cl (mmol/L)	105.81 ± 4.59	106.52 ± 5.09	0.44
	CO2CP (mmol/L)	21.38 ± 3.94	21.19 ± 5.44	0.83
	Ca (mmol/L)	2.15 ± 0.25	2.15 ± 0.19	0.99
	IP (mmol/L)	1.16 ± 0.23	1.26 ± 0.4	0.09
Nutrition index	Alb (g/L)	36.9 (32.95, 41.68)	34.55 (30.50, 38.08)	0.28
	Fe (umol/L)	17.53 ± 8.69	15.61 ± 9.9	0.28
	Transferrin (g/L)	2.09 ± 0.63	1.9 ± 0.65	0.13
	PA (g/L)	244.36 ± 74.48	265.64 ± 94.59	0.22
Inflammatory index	CP (mg/L)	330.37 ± 188.62	326.6 ± 137.53	0.91
	ESR (mm/hr)	31 (21, 57)	58.50 (30.50, 73.50)	0.06
	CRP (mg/L)	1.9 (0.4, 7.36)	1.40 (0.70, 5.18)	0.71
Glomerular function	s*α*1-MG (mg/L)	55.04 ± 20.58	55.71 ± 23.93	0.87
	s*β*2-MG (mg/L)	6.22 ± 3.41	8.42 ± 6.49	0.021
	sRBP (mg/L)	60.9 ± 81.13	51.36 ± 56.26	0.50
	BF (mg/L)	323.37 ± 95.19	292.68 ± 164.87	0.21
	Hcy (umol/L)	19.65 (11.75, 24.58)	17.60 (9.75, 26.40)	0.16
	sNAG (U/L)	24.95 ± 28.21	23.57 ± 13.55	0.77
Renal tubular function	u*β*2-MG (*μ*g/mL)	0.63 (0.15, 4.43)	2.20 (1.06, 4.44)	0.52
	umicroAlb (mg/L)	264.1 (85.6, 631.5)	267.20 (138.15, 572.25)	0.89
	u*α*1-MG (*μ*g/mL)	14.45 (8.55, 24.88)	19.80 (9.15, 39.70)	0.033
	uRBP (mg/L)	6.13 (1.87, 9.48)	6.99 (2.97, 13.00)	0.49
	uNAG (U/L)	9.4 (5.1, 17.75)	8.50 (4.75, 19.20)	0.79
uACR (mg/L)		39.34 (8.63, 106.67)	80.73 (19.25, 154.36)	0.036
Kidney size (cm^3^/m^2^)		162.58 ± 72.27	149.71 ± 64.91	0.035
Renal parenchyma	I	3 (4.3%)	2 (4.8%)	0.037
	II	25 (35.7%)	8 (19%)	
	III	7 (10%)	6 (14.3%)	
	IV	35 (50%)	26 (61.9%)	
CTR		0.51 ± 0.05	0.53 ± 0.06	0.049

Alb: albumin; BF: B factor; Ca: Calcium; Cl: chlorine; CO2CP: carbon dioxide binding force; CP: Copper blue protein; CTR: cardiothoracic ratio; CysC: cystatin C; eGFR: estimated glomerular filtration rate; Hb: hemoglobin; Hct: hematocrit; Hcy: homocysteine; HDL-C: high-density lipoprotein cholesterol; IP: phosphorus; K: potassium; LDL-C: low-density lipoprotein cholesterol; Na: sodium; PA: prealbumin; S *α*1-MG: blood *α*1 microglobulin; S *β*2-MG: blood *β*2 microglobulin; SRBP: Schromatol protein; TC: total cholesterol ester; TG: total triglycerides; UA: uric acid; UACR: ratio of urinary albumin creatinine; umicroAlb: urinary microalbumin; USG: urine gravity; U*α*1-MG: urine *α*1 microglobulin; U*β*2-MG: urine *β*2 microglobulin; WBC: white blood cell count.

**Table 3:** Independent risk factors for rapid progression of renal function in patients with PGD.

Index	B	*P* value	OR	95% CI for OR	
CTR	1.559	0.004	5.943	1.185	9.802
SBP	0.042	0.006	1.043	1.012	1.075
HDL	-1.620	0.024	0.198	0.048	0.817
Kidney volume	-0.007	0.042	0.907	1.000	1.013

**Table 4:** Baseline and follow-up case of not rapid progression group and rapid progression group.

Variates		Baseline	Endpoint	*t*/*x*^2^	*P*
CTR	NR group	0.51 ± 0.04	0.51 ± 0.038	2.172	0.05
	R group	0.53 ± 0.065	0.58 ± 0.054^a^	3.176	0.005
SBP	NR group	130.23 ± 17.2	129.75 ± 17.21	1.200	0.24
	R group	140.33 ± 16.8	148.42 ± 18.31^a^	3.030	0.005
HDL	NR group	1.24 (0.94, 1.44)	1.25 (0.98, 1.5)	-1.736	0.09
	R group	1.21 (0.91, 1.34)	1.1 (0.96, 1.19)^a^	4.534	0.005
Kidney volume	NR group	162.19 ± 83.53	157.99 ± 83.39	0.673	0.51
	R group	175.78 ± 53.84	134.60 ± 74.24	3.033	0.007

^a^Compared with the group NR at the follow-up endpoint, *P* < 0.05.

**Table 5:** Baseline and follow-up case of indicators in different CKD stage.

Index	CKD stage 3 (*n* = 52)		
	Baseline	Follow-up endpoint	*P* value
CTR	0.52 ± 0.05	0.53 ± 0.046	0.028
SBP	129.78 ± 14.77	136.76 ± 19.70	0.035
HDL	1.33 (1.04, 1.55)	1.31 (1.03, 1.44)	0.043
Kidney volume	156.33 ± 59.62	145.56 ± 52.94	0.21
Index	CKD stage 4 (*n* = 60)		
	Baseline	Follow-up endpoint	*P* value
CTR	0.51 ± 0.054	0.53 ± 0.05	0.008
SBP	136.42 ± 17.15	140.49 ± 22.72	0.049
HDL	1.07 (0.89, 1.28)	1.05 (0.84, 1.15)	0.037
Kidney volume	185.32 ± 83.02	151.99 ± 99.85	0.019

## Data Availability

All data are available upon request to correspondence author.
